# Fixating on metals: new insights into the role of metals in nodulation and symbiotic nitrogen fixation

**DOI:** 10.3389/fpls.2014.00045

**Published:** 2014-02-13

**Authors:** Manuel González-Guerrero, Anna Matthiadis, Áez;ngela Sáez, Terri A. Long

**Affiliations:** ^1^Centro de Biotecnología y Genómica de Plantas, Universidad Politécnica de MadridMadrid, Spain; ^2^Department of Plant and Microbial Biology, North Carolina State UniversityRaleigh, NC, USA

**Keywords:** symbiotic nitrogen fixation (SNF), metals, legume, rhizobia, nodule, iron, zinc, copper

## Abstract

Symbiotic nitrogen fixation is one of the most promising and immediate alternatives to the overuse of polluting nitrogen fertilizers for improving plant nutrition. At the core of this process are a number of metalloproteins that catalyze and provide energy for the conversion of atmospheric nitrogen to ammonia, eliminate free radicals produced by this process, and create the microaerobic conditions required by these reactions. In legumes, metal cofactors are provided to endosymbiotic rhizobia within root nodule cortical cells. However, low metal bioavailability is prevalent in most soils types, resulting in widespread plant metal deficiency and decreased nitrogen fixation capabilities. As a result, renewed efforts have been undertaken to identify the mechanisms governing metal delivery from soil to the rhizobia, and to determine how metals are used in the nodule and how they are recycled once the nodule is no longer functional. This effort is being aided by improved legume molecular biology tools (genome projects, mutant collections, and transformation methods), in addition to state-of-the-art metal visualization systems.

## INTRODUCTION

Substantial effort has been put forth to increase legume growth and production of seeds with enhanced nutritional content and bioavailability. In non-legume crops, investigators have used plant breeding and biotechnology to successfully increase metal uptake from soil and delivery to the shoot (Bashir et al., 2013). However, in legumes, in addition to the metabolic processes common to all plants, metals are also required the unique process of symbiotic nitrogen fixation (SNF). SNF heightens the demand for these same metal nutrients in legumes, often exhibiting signs of nutritional stress when undergoing nodulation and SNF (Terry et al., 1991; Slatni et al., 2012). Moreover, nodules, specialized root structures in which SNF occurs, and the process of nodulation itself, increase sensitivity to metal nutrient availability. This review highlights early and more recent studies that provide insight into the role of the nutritional metals in the various stages of the rhizobium–legume symbiosis.

## STAGES OF NODULE DEVELOPMENT

Nodulation begins with the recognition of host plant-induced rhizobial nod factors by receptors within the membranes of plant root epidermal cells. This triggers calcium oscillations, membrane depolarization, and signal transduction cascades that lead to root hair curling and root cell wall degradation at the site of infection (for review, see Oldroyd and Downie, 2004). Rhizobia can then enter the root via invagination of the epidermal cell plasma membrane, forming an infection thread that grows and eventually releases rhizobia into the cytosol of nodule primordial cells within the root cortex. The rhizobia and their surrounding peribacteroid membrane (PBM), together known as the symbiosome, continue to divide until cells are filled with thousands of symbiosomes (for review, see Udvardi and Poole, 2013). Eventually, the bacteria within the symbiosomes stop dividing and differentiate into nitrogen-fixing bacteroids.

Many legumes from tropical and subtropical regions (soybean, bean) and some from temperate regions (*Lotus japonicus*) develop determinate nodules, in which meristem activity halts, causing the formation of a spherical nodule. In contrast, most other temperate legumes, including those in the genera *Medicago*, *Trifolium*, and *Pisum*, develop indeterminate nodules. Indeterminate nodules maintain a nodule meristem at the growing tip (zone I), followed by a zone of infection where rhizobia are released from the infection thread (zone II), a nitrogen fixation zone (zone III), and finally, a senescence zone in which bacteroids are degraded and nitrogen fixation ceases (zone IV; Vasse et al., 1990). Nutrient exchange at the nodule is facilitated by vascular vessels that surround cortical infected and uninfected cells.

## METAL FUNCTIONS IN THE ESTABLISHMENT OF SNF

Metals are key elements of all living organisms (Frausto da Silva and Williams, 1991) and are an integral part of 30–50% of the proteome of a typical cell (Waldron and Robinson, 2009). They are also involved in every biological process, including the legume-specific stages of SNF from rhizobia infection to nodule senescence. During the initial stages of infection, manganese and calcium facilitate rhizobial colonization of the root by mediating rhizobial lectin binding to the root hair tips (Kijne et al., 1988). Later stages of SNF signaling are mediated by calcium-spiking in the perinuclear region. As a result, calcium-calmodulin dependent kinases (CCaMKs) are activated (Singh and Parniske, 2012). CCaMKs induce the expression of genes mediating nodulation via the transcription factors NSP1/2 or NIN (Kalo et al., 2005; Andriankaja et al., 2007). Additionally, high levels of potassium have been detected in the apical region of indeterminate nodules, where it might play a role in cell growth (Rodríguez-Haas et al., 2013). Potassium also acts as the counter-ion compensating calcium in earlier signaling processes, via transport processes putatively mediated by channels such as CASTOR, POLLUX or DMI1 (Peiter et al., 2007; Charpentier et al., 2008, 2013). Also, cobalt, a component of cobalamin in ribonucleotide reductases, is essential for rhizobia endoreduplication, which occurs during differentiation into bacteroids (Taga and Walker, 2010).

Iron is the key cofactor of many metabolic reactions involved in SNF. In the early stages of nodulation, heminic iron is critical for catalase-mediated free radical detoxification (Jamet et al., 2003). Upon nodule maturation, iron is required for nitrogenase and leghemoglobin activity. The bacterial nitrogenase complex is necessary for the actual production of a usable nitrogen source (ammonia) from atmospheric nitrogen. NifH and NifDK, components of the nitrogenase complex, require iron–sulfur clusters and the iron–molybdenum cofactor, respectively. In contrast, leghemoglobin, the most abundant protein in the nodule cytosol, contains heminic iron. It effectively buffers oxygen content in the nodule, allowing for levels sufficient for bacteroid respiration without inactivating nitrogenase. Heminic iron is also critical for energy transduction by rhizobia cytochromes (Reedy and Gibney, 2004). Free iron, directly coordinated by amino acids, is involved in free radical detoxification as part of Fe-superoxide dismutases (Fe-SOD; Rubio et al., 2007).

Copper also plays a critical role in nitrogen fixation. Increased copper application results in elevated nitrogen fixation per nodule and increased nitrogen content in plant tissue (Hallsworth et al., 1960; van der Elst et al., 1961; Snowball et al., 1980; Seliga, 1990, 1993). This element is a cofactor of some of the high affinity cytochromes mediating energy transduction in the bacteroid (Preisig et al., 1996). It is also part of several superoxide dismutase systems detoxifying abundant free radicals produced as side products of SNF (Rubio et al., 2007). Additionally, zinc and manganese are integral elements of many superoxide dismutases (Rubio et al., 2007). Zinc is also involved in gene regulation as part of the zinc-finger motif of many transcription factors (Klug, 2010).

Some rhizobia are able to conserve energy by oxidizing H_2 _ produced during nitrogen fixation via the uptake hydrogenase enzyme, Hup (Emerich et al., 1979). Hup contains nickel, and alterations in nickel supply have been shown to affect Hup activity and formation in soybean and pea (Klucas et al., 1983; Stults et al., 1984; Brito et al., 1994, 1997). Therefore, although not required for nitrogen fixation, nickel can enhance the effectiveness of SNF in those species containing hydrogenase-encoding species, and may prove more critical if breeding and engineering efforts focused around this enzyme are successful.

## TRANSITION METAL UPTAKE AND DISTRIBUTION IN SNF

**FIGURE 1:**
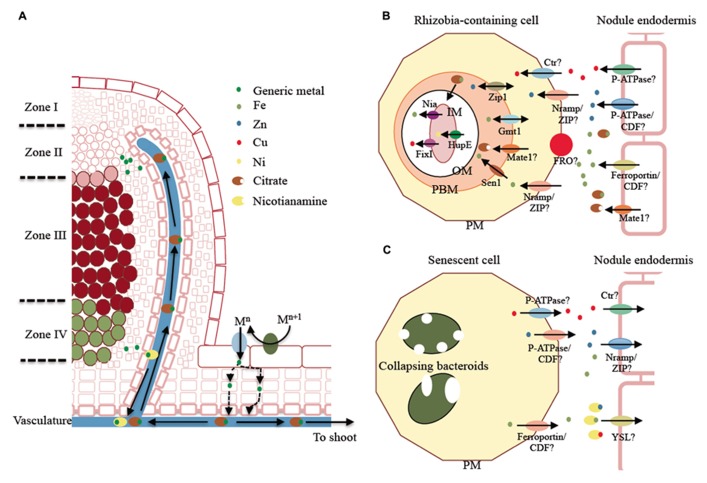
**Model of transition metal transport in indeterminate type nodules.** **(A)** Overview of the metal delivery mechanism. Metals are typically incorporated following Strategy I, i.e., reduction by a plasma membrane ferroreductase and uptake by plasma membrane metal transporters. This process occurs over the entire epidermal surface of the root, including the nodule. Metals reach the vasculature where they bind metal chelators as citrate. The metal citrate complex is delivered to zone II of the nodule (infection/differentiation zone) and the metal is released into the apoplast and subsequently incorporated into the infected cell. In zone IV (senescence zone), metals are released to the apoplast, associated with nicotianamine, and translocated into the vasculature. **(B)** Proposed model of metal delivery from the vasculature to zone II. Proposed transporter families to carry out ion transport are indicated with a question mark. **(C)** Proposed model of metal recovery from the senescent zone of the nodule. Proposed transporter families are indicated with a question mark. PM indicates plasma membrane; PBM, peribacteroid membrane; OM, bacterial outer membrane; and IM, bacterial inner membrane.

Transition metal transport processes in the nodule are summarized in **Figure 1**. Legumes are Strategy I plants (Andaluz et al., 2009), i.e., iron is incorporated after acidification of the soil, which increases Fe^3^^+^ solubility. Ferroreductase can then reduce Fe^3^^+^ to Fe^2^^+^, which is finally transported into the epidermal cell by ZIP or NRAMP family members (Curie et al., 2000; Morrissey and Guerinot, 2009; **Figure 1A**). Copper is likely incorporated in a similar manner. Cu^2^^+^ is reduced to Cu^+^ and a member of the copper transporter (Ctr) family translocates the metal across the epidermal cell plasma membrane. Zn^2^^+^ and other divalent ions are possibly directly introduced by ZIP or NRAMP family members. Many dicots will also secrete organic acids, phenolics, flavins, and flavonoid compounds under iron deficiency (Rodríguez-Celma et al., 2013). Phenolic compounds have recently been shown to modify the rhizosphere microbial community, leading to increased synthesis of the metal chelators and siderophores in red clover (Jin et al., 2006), which may also facilitate root metal uptake.

Symbiotic nitrogen fixation exerts a heavy toll on plant metal reserves, eliciting up-regulation of metal uptake systems (Terry et al., 1991). Recently, Slatni et al. (2011) measured the overall contribution of nodule versus root in rhizospheric iron uptake, noticing increased ferroreductase activity in the nodule. This could be due to the nodule epidermis playing a major role in iron uptake. Alternatively, since the ferroreductase activity was measured in the microsomal fraction of nodule extracts, some of this increased activity could result from ferroreductases working within the nodule cortex. This alternative hypothesis would take into account that the Fe^3^^+^, transported as a citrate complex (Rellán-Álvarez et al., 2010), would have to be reduced to Fe^2^^+^ in the nodule cortex in order to be incorporated by the vasculature cells. In addition, H^+^-ATPases and ZIP family members are expressed in the epidermal layer, in close proximity to the vasculature, and in the nitrogen fixation zone of the nodule (Slatni et al., 2012). These observations indicate that iron is present in the apoplast of the nodule cortex and, therefore, cortical cells use these proteins to uptake apoplastic iron.

Synchrotron-based X-ray fluorescence (S-XRF) studies indicate that some legumes with indeterminate nodules deliver iron, and likely other metals, to the nodule through the vasculature rather than using an epidermal pathway (Rodríguez-Haas et al., 2013). The metal distribution throughout different regions of the nodule indicates that there is a massive accumulation of iron around the vascular cells in zone II extending into the nodule cortical cells. Interestingly, this is where symbiosis is established and the symbiosomes differentiate. Iron accumulation in the apoplast would require plasma membrane metal transporters to introduce this element into the cytosolic compartment to synthesize ferroproteins. This transport system seems to be very efficient, since very little or no apoplastic iron is observed in the nitrogen fixation zone of the nodule. Researchers speculate that a ZIP or NRAMP transporter, both involved in divalent metal uptake (Nevo and Nelson, 2006; Lin et al., 2010), are the likely candidates (**Figures 1A,B**).

ZIP family members have been immunodetected in the nodule (Slatni et al., 2012). However, the only ZIP family member characterized in this organ, GmZIP1, seems to be localized in the PBM (Moreau et al., 2002), where it is predicted to play a role in Zn^2^^+^ transport. In this membrane, an Fe^2^^+^-transporting NRAMP member, GmDMT1, is also present (Kaiser et al., 2003; **Figure 1B**). These transporters may be involved in metal transport to the symbiosome. However, biochemical analysis of members of these two families and yeast complementation studies indicate that neither transporter could play a role in metal transfer to the symbiosome (Nevo and Nelson, 2006; Lin et al., 2010), instead they appear to transport metals towards the cytosol. The localization observed could be the result of the endocytic process mediating rhizobia release into the host cell (Leborgne-Castel et al., 2010), which would also carry plasma membrane associated transporters.

A more likely candidate for iron transport into the symbiosome is SEN1 (**Figure 1B**). The *sen1* mutant in *L. japonicus *has a number of alterations in nodulation that are associated with the lack of nitrogenase activity, most likely due to a deficiency in iron loading of the symbiosomes (Hakoyama et al., 2012). The proposed role of SEN1 in iron transport is due to its close sequence similarity to *Saccharomyces cerevisiae* CCC1 and *Arabidopsis thaliana* VIT1 proteins, both involved in divalent metal ion transport into organelles (Li et al., 2001; Kim et al., 2006). However, more detailed analyses, such as subcellular localization of the transporter, characterization of iron distribution, or the restoration of the phenotype by the addition of external iron, would be required to conclude this with certainty

Once metals cross the PBM, they are incorporated and used by the bacteroid. However, in spite of the huge number of genomic sequences available from rhizobia, very little is known about which transporters are involved in metal uptake and usage (**Figure 1B**). One of the first studies indicates that a P_1b_-type Cu^+^-ATPase, FixI, is essential for nitrogen fixation (Kahn et al., 1989). FixI is responsible for transporting Cu^+^ to the bacteroid periplasm. Within this compartment Cu^+^ is integrated into membrane-bound cytochrome cbb3 oxidase (Preisig et al., 1996), which is responsible for energy transduction in microaerobic environments. The Ni^2^^+^ importers HupE1 and HupE2 play a similar role in providing metal for the assembly of the Ni–Fe cofactor of hydrogenase (Brito et al., 2010). No direct evidence for an iron importer is available, but there is evidence of protective mechanisms against the local accumulation of toxic concentrations of this element. For example, the P_1b_-type ATPase, Nia, is responsible for detoxifying excess Fe^2^^+^ that accumulates upon the massive entry of iron utilized to synthesize nitrogenase and other ferroproteins (Zielazinski et al., 2013).

The role of citrate in iron transport is important, although its role in SNF has not been completely elucidated. The citrate transporter FRD3, a multidrug and toxic compound extrusion (MATE) protein family member, has been shown in *A. thaliana* to be essential for iron transport across symplastically disconnected tissues (Roschzttardtz et al., 2011). Differences in the expression of citrate transporter *GmFRD3 *is a major contributing factor that distinguishes iron efficient soybean cultivars from iron inefficient cultivars. This finding suggests that iron efficient varieties exhibit increased tolerance to low iron due to increased solubility of ferric iron, which is facilitated by increased xylem citrate (Rogers et al., 2009). However, the function of GmFRD3 has not been examined in nodulating roots, therefore the role of this putative transporter within the context of SNF is still unknown. At the PBM a citrate transporter is also likely be important, since it has been shown that some rhizobia have a preference for citrate as their siderophore (LeVier et al., 1996). Takanashi et al. (2013) recently reported nodule-specific expression of a* L. japonicus* MATE family member, LjMATE1 (**Figure 1B**). LjMATE1 appears to have a substantial effect on iron distribution and nitrogenase activity in this organ (Takanashi et al., 2013). However, no precise localization of this transporter has been provided to date, and as a result the role of this transporter (long distance iron transport versus PBM translocation) could not be discerned.

## NODULE SENESCENCE AND SEED SET

Nodule senescence is a programmed process coupled with the entry into the reproductive stage of the host plant life cycle (Van de Velde et al., 2006). Given that iron is a growth-limiting nutrient (Grotz et al., 1998), it has to be recycled from the senescent nodule. A number of studies indicate that this is the case (Burton et al., 1998; Rodríguez-Haas et al., 2013; **Figure 1C**). In young plants, some of this recycled iron might be redirected to younger parts of the nodule, but it would be predominantly transported to the shoot through the vasculature as the plant enters its reproductive stage. Burton et al. (1998) estimated that around 50% of the total nodular iron is recycled in the seed, in a process that is likely to be reminiscent of leaf senescence (Shi et al., 2012). Although no senescence-upregulated metal transporter has been identified, a senescent nodule-specific nicotianamine (NA) synthase has been cloned (Hakoyama et al., 2009). The synthesis of NA, the molecule responsible for intracellular and phloem metal transport (Curie et al., 2009), indicates that the released metals are transported within the phloem using an unknown Yellow Stripe-like (YSL) transporter, since YSLs are responsible for NA-metal transport (Curie et al., 2009).

The steps leading to cell death during senescence include degradation of plant tissue via free radical oxidation (Thompson et al., 1987). Free radical production *in planta* can be catalyzed by transition metals in the Fenton reaction (Stohs and Bagchi, 1995). Given the high concentration of iron in the nodule, it is likely that it is responsible for accelerating free radical production and eventual senescence (Bhattacharjee, 2005), as evidenced by the strong reduction in nodular deoxyribose degradation and linolenic acid peroxidation in the presence of the iron chelator, desferrioxamine (Becana and Klucas, 1992). No evidence exists for the involvement of other metals in this process, especially given the fact that concentrations of other catalytic metals are likely too low to contribute.

## FUTURE DIRECTIONS

Although we have learned a great deal about the developmental and signaling processes involved in SNF in recent years, many questions remain about the molecular mechanisms by which nutrients, metals in particular, are transported to and from developing and mature nodules. Recent advances in high-resolution elemental analysis have been used to show changes in iron localization in indeterminate nodules (Rodríguez-Haas et al., 2013). Continued use of these and other elemental localization techniques (for review, see Samira et al., 2013; Zhao et al., 2014) such as energy-dispersive X-ray analysis or nanoSIMS to detect other elements should facilitate great strides in understanding metal distribution and translocation in SNF in the near future.

Recent transcriptomic approaches have also been particularly useful for identifying whole-genome responses involved in nutrient translocation and assimilation during various developmental stages of SNF. Transcriptional profiles at the onset of *Mesorhizobium loti* infection, during nodule primordia initiation and nodule organogenesis, and at the onset of nitrogen fixation indicate that there is little overlap between transcripts present at the earlier stages of infection and those present in fully developed nodules (Takanashi et al., 2012). This same study led to the discovery of LjMATE1, the putative dicarboxylic acid (citrate) transporter described above. Additionally, transcripts involved in the transport of carbohydrates, metals, and peptides are abundant at all stages of nodule development. Transcriptomic analysis of *L. japonicus* nodules of primary and lateral roots, followed by non-biased metabolic profiling, revealed that the majority of nodule-specific genes are involved in carbon and amino acid metabolism, while 5% are involved in transport of metabolites or inorganic ions (Colebatch et al., 2004). Molecular genetic analysis of candidate genes identified by these studies should greatly enhance our understanding of regulatory processes that facilitate nutrient transport for SNF.

Overall, few studies have examined genotypic difference in response to metal availability in the context of SNF. So the question remains: what part of the changes in the nodule metallo-transcriptome are metal dependent and which are symbiosis-dependent? Time course and split root experiments of nodulating and non-nodulating roots under nutrient-sufficient and -deficient conditions followed by cell-specific transcriptional, proteomic, and metabolic profiling could begin to answer the question above. Furthermore, the application of modeling approaches and comparative studies will allow for the identification of metal homeostasis factors that are specific to symbiotic interactions and enable efforts for increased production of leguminous crops.

## Conflict of Interest Statement

The authors declare that the research was conducted in the absence of any commercial or financial relationships that could be construed as a potential conflict of interest.
